# Psychological determinants of physical activity across the life course: A "DEterminants of DIet and Physical ACtivity" (DEDIPAC) umbrella systematic literature review

**DOI:** 10.1371/journal.pone.0182709

**Published:** 2017-08-17

**Authors:** Cristina Cortis, Anna Puggina, Caterina Pesce, Katina Aleksovska, Christoph Buck, Con Burns, Greet Cardon, Angela Carlin, Chantal Simon, Donatella Ciarapica, Giancarlo Condello, Tara Coppinger, Sara D’Haese, Marieke De Craemer, Andrea Di Blasio, Sylvia Hansen, Licia Iacoviello, Johann Issartel, Pascal Izzicupo, Lina Jaeschke, Martina Kanning, Aileen Kennedy, Fiona Chun Man Ling, Agnes Luzak, Giorgio Napolitano, Julie-Anne Nazare, Grainne O’Donoghue, Camille Perchoux, Tobias Pischon, Angela Polito, Alessandra Sannella, Holger Schulz, Rhoda Sohun, Astrid Steinbrecher, Wolfgang Schlicht, Walter Ricciardi, Loriana Castellani, Ciaran MacDonncha, Laura Capranica, Stefania Boccia

**Affiliations:** 1 Department of Human Sciences, Society, and Health, University of Cassino and Lazio Meridionale, Cassino, Italy; 2 Section of Hygiene—Institute of Public Health; Università Cattolica del Sacro Cuore, Rome, Italy; 3 Department of Movement, Human and Health Sciences, University of Rome Foro Italico, Rome, Italy; 4 Leibniz Institute for Prevention Research and Epidemiology, BIPS, Bremen, Germany; 5 Department of Sport, Leisure and Childhood Studies, Cork Institute of Technology, Cork, Munster, Ireland; 6 Department of Movement and Sports Sciences, Ghent University, Ghent, Belgium; 7 Department of Physical Education and Sport Sciences, Health Research Institute, University of Limerick, Limerick, Ireland; 8 Centre de Recherche en Nutrition Humaine Rhône-Alpes, Univ-Lyon, CarMeN Laboratory, INSERM 1060, INRA 1397, Université Claude Bernard Lyon1, Lyon, France; 9 Council for Agricultural Research and Economics—Research Centre for Food and Nutrition, Rome, Italy; 10 Department of Medicine and Aging Sciences, 'G. d'Annunzio' University of Chieti-Pescara, Chieti, Italy; 11 Department for Sport and Exercise Sciences, Division of Exercise and Health Sciences I, University of Stuttgart, Stuttgart, Germany; 12 Department of Epidemiology and Prevention, IRCCS Istituto Neurologico Mediterraneo NEUROMED, Pozzilli, Italy; 13 Department of Medicine and Surgery, University of Insubria, Varese, Italy; 14 School of Health and Human Performance, Multisensory Motor Learning Lab., Dublin City University, Dublin, Ireland; 15 Max Delbrück Center for Molecular Medicine (MDC), Berlin, Germany; 16 Department of Sport Sciences, Social and Health Sciences, University of Konstanz, Konstanz, Germany; 17 Centre for Preventive Medicine, School of Health and Human Performance, Dublin City University, Dublin, Ireland; 18 Institute of Sport, Exercise & Active Living, Victoria University, Melbourne, Australia; 19 Department of Psychology, Faculty of Science & Technology, Bournemouth University, Bournemouth, United Kingdom; 20 Institute of Epidemiology I, Helmholtz Zentrum München, German Research Center for Environmental Health, Neuherberg, Germany; 21 Luxembourg Institute of Socio-Economic Research, Esch/Alzette, Luxembourg; 22 Charité Universitätsmedizin Berlin, Berlin, Germany; 23 DZHK (German Center for Cardiovascular Research), partner site Berlin, Berlin, Germany; 24 Italian National Institute of Health (Istituto Superiore di Sanità—ISS), Rome, Italy; 25 Icahn School of Medicine at Mount Sinai, New York, NY, United States of America; Vanderbilt University, UNITED STATES

## Abstract

Low levels of physical activity (PA) are reported to contribute to the occurrence of non-communicable diseases over the life course. Although psychological factors have been identified as an important category concerning PA behavior, knowledge on psychological determinants of PA is still inconclusive. Therefore, the aim of this umbrella systematic literature review (SLR) was to summarize and synthesize the scientific evidence on psychological determinants of PA behavior across the life course. A systematic online search was conducted on MEDLINE, ISI Web of Science, Scopus, and SPORTDiscus databases. The search was limited to studies published in English from January 2004 to April 2016. SLRs and meta-analyses (MAs) of observational studies investigating the association of psychological variables and PA were considered eligible. Extracted data were evaluated based on importance of determinants, strength of evidence, and methodological quality. The full protocol is available from PROSPERO (Record ID: CRD42015010616). Twenty reviews (14 SLRs and 6 MAs), mostly of moderate methodological quality, were found eligible. Convincing evidence was found for self-efficacy (positive association with PA) in children and adolescents, and stress (negative association with PA) regardless of age. Most of the evidence revealing an association between psychological determinants and PA is probable and limited, mainly due to differences in the definition of PA and of psychological determinants across reviews. Thus, scholars are urged to reach a consensus on clear definitions of relevant psychological determinants of PA, subsuming cultural biases and allowing the possibility to obtain clear interpretations and generalizability of findings. Finally, most psychological determinants should be considered within a larger framework of other multi-level determinants that may interact or mediate some of the effects.

## Introduction

Physical activity (PA) is a health enhancing behavior that is effective at reducing the risk of a range of non-communicable diseases such as obesity, cancer, type II diabetes, hypertension, and chronic cardiovascular and respiratory diseases [1]. Although the European Union (EU) is strongly engaged in promoting health-enhancing PA [2] for all individuals independently from age and social status, a large proportion of the population are fail to meet these guidelines, with approximately one third of adults (31%) and the majority of young people aged 13–15 years (80%) worldwide classed as physically inactive [3], thus exposing themselves to health risk.

Several models for the exploration of the active lifestyle choices have been proposed [4–8], looking at individual (biological, psychological, and behavioral aspects), interpersonal (relationships with parents, relatives, peers, and socio-cultural networks), environmental (access/availability of tools/services, and proximal/distal built/natural surroundings), and policy (organizational and governmental aspects) dimensions. Within those dimensions, positive, negative, inconclusive, or no associations might exist between several determinants and PA. However, both determinants and PA present a great diversity in research designs, measurement approaches, populations studied, types of measurement and terminologies, which still make difficult to draw a comprehensive understanding. In general, the term ‘determinant’ is used to address causal variables also including correlates (i.e., multiple variables intervening in cause-effect relationships), whilst mediators (i.e., variables influencing a cause-effect relationship between variables), moderators (i.e., variables effecting the strength of a relationship between variables), and/or confounders (i.e., variables associated with the outcome that distort the observed relationships) are considered different variables [9,10]. According to a review approach that is acquiring relevance in public health as a mean to complement systematic and meta-analytic review modes—the realist synthesis approach—determinants are similar to mechanisms (i.e., an idea about what works to change a given behavior in an expected direction under given circumstances and why it works [11]). Furthermore, a lack of commonality exists in the PA terminology applied in the studies and different forms of PA are considered, ranging from unstructured daily activities, occupational PA, leisure time PA to structured PA (e.g., exercise, grassroots sports, and competitive sports) and considering the most relevant parameters of PA, such as frequency (e.g., daily, weekly, monthly), duration (e.g., total time of activity, rest intervals), and intensity (e.g., low, moderate, moderate-vigorous, vigorous, maximal efforts).

Although there are several ways in which PA behaviors could be conceptualized and defined and different factors may influence individual choices [12], psychological factors are direct determinants of maintenance of PA [4]. Therefore, a greater understanding of the determinants of involvement in exercise (Ex) and PA, including motivation, seems to be necessary [13]. In particular, descriptive research on participation suggested fun, skill development, affiliation, fitness, success and challenge for youth; challenge, skill development and fitness for adults; health, relaxation and enjoyment for older individuals, as motives to reflect involvement in sport, Ex and PA. Also self-efficacy, attitude, intentions and perceived physical competence seem to be significant predictors of PA adherence and compliance [13–15]. On the other hand, issues of safety and feelings of incompetence are reported as perceived barriers [13].

Despite the attempts made to clarify the psychological determinants of PA, sound knowledge and understanding of how and why people adopt and/or maintain adequate PA levels, as well as systematic analysis, are still missing. The diversity in research designs, theoretical and measurement approaches, population groups, determinants investigated, and PA outcomes, across the literature, makes it difficult to understand the evidence and to draw appropriate conclusions on the importance of psychological determinants in influencing PA behaviors [13].

Recently, the European Commission endorsed a Joint Programming Initiative (JPI) to increase research capacity across Member States to engage in a common research agenda on healthy diet and healthy lifestyles [16] and the DEterminants of DIet and Physical Activity-Knowledge Hub (DEDIPAC-KH) [17]. To expand knowledge and to develop new insights and initiatives to promote PA, the DEDIPAC-KH organized and carried out an umbrella systematic literature review (SLR) [18] on all the possible determinants of PA. Overall, 7 categories of determinants of PA have been identified: biological, psychological, behavioral, physical, socio-cultural, socio-economic, and policy. Due to the amount of researches available, the DEDIPAC-KH Management Team decided to organize the findings in 7 separated umbrella SLRs, each focused on a single category. The DEDIPAC-KH Management Team is conscious that splitting the categories may cause a lost in the analysis of the interactions between those categories of determinants which may share commonalities. However, this strategy was considered necessary for a clear dissemination of insights on the determinants of PA.

Therefore, the aim of this umbrella SLR is to give an overview of the evidence on psychological determinants of PA by systematically reviewing the available evidence from existing SLRs and meta-analyses (MAs) of primary observational studies.

## Materials and methods

The manuscript was drafted following the PRISMA checklist [19], provided in S1 Checklist. A common protocol for the seven umbrella SLRs was registered and is available on PROSPERO (Record ID: *CRD42015010616*), the international prospective register of systematic reviews [20]. Review title, timescale, team details, methods, and general information were recorded in the PROSPERO register prior to completing data extraction.

### Search strategy and eligibility criteria

**Fig 1:**
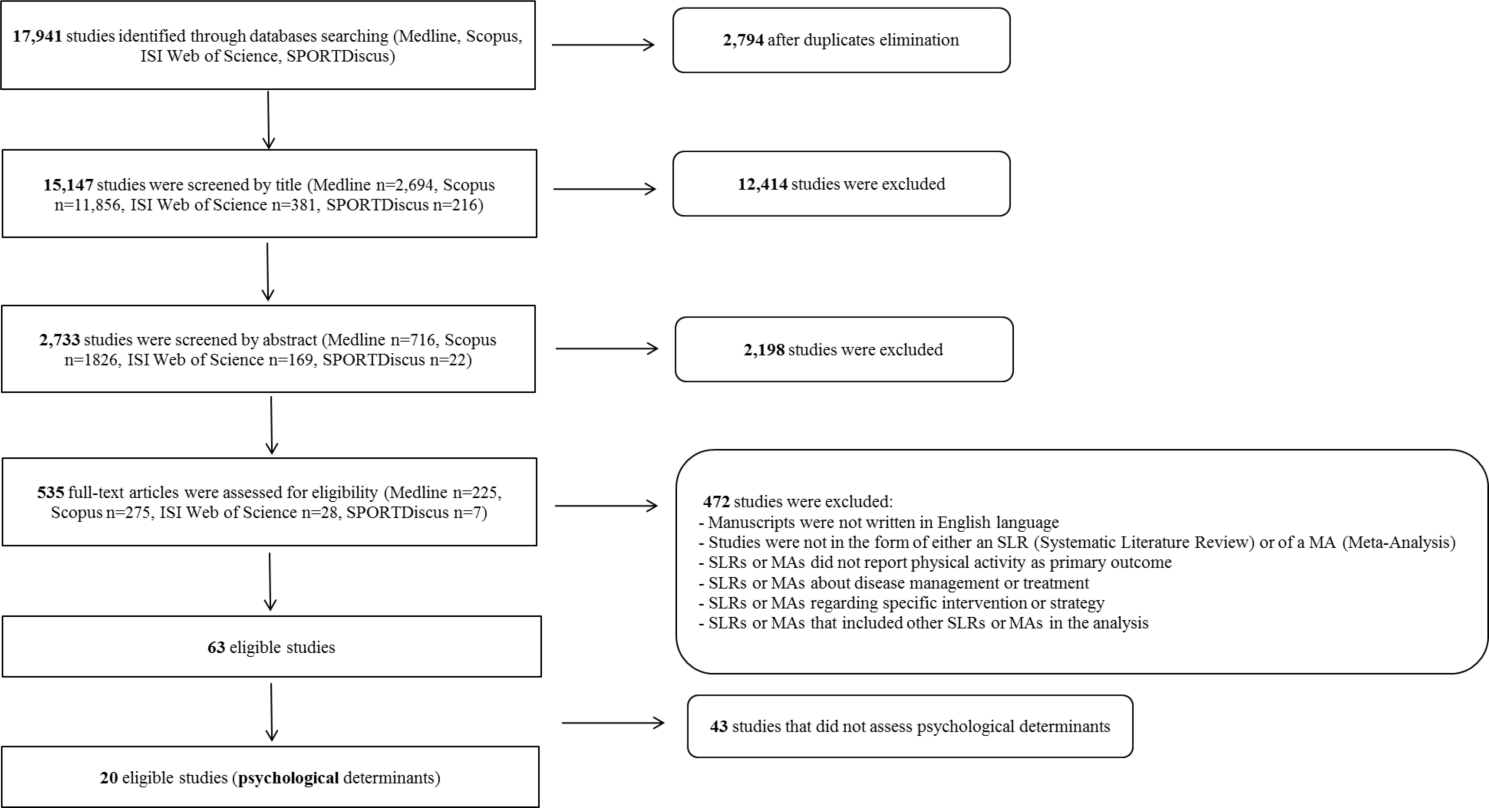
Flowchart of the literature research by database.

SLRs and MAs investigating the determinants of PA across the life course were systematically searched on MEDLINE, ISI Web of Science, Scopus, and SPORTDiscus databases. The search was limited to SLRs and MAs published in English, between January, 1^st^ 2004 and April, 30^th^ 2016. SLRs and MAs published before 2004 were not included to avoid duplications of the earliest individual studies included in the SLRs and MAs. According to the literature [21], Table 1 shows the MEDLINE search strategy, and Fig 1 summarizes the process of the literature research, common to the subsequent 7 umbrella SLRs. Thus, Table 1 provides the overall list of searched terms, whilst Fig 1 shows the count of the overall excluded/included reviews, related and not-related with psychological determinants.

**Table 1:** Search strategy: Key words used for the literature research.

Set	Search terms
#1	“physical activit*” OR “physical exercise*” OR sport OR “motor activit*” OR “locomotor activit*” OR athletic* OR fitness OR “physical movement*” OR “physical performance*” OR “aerobic exercise*” OR “physical effort*” OR “physical exertion*”
#2	determinant OR determinants OR correlator OR correlators OR mediator OR mediators OR moderator OR moderators OR contributor OR contributors OR factor OR factors OR association OR modifier OR modifiers OR confounder OR confounders OR pattern OR patterns OR predictor*
#3	demographic* OR motivation OR cognition OR emotion* OR attitude* OR “self-perception” OR “self-confidence” OR “self-efficacy” OR competence OR reward* OR success* OR challenge* OR knowledge OR belief* OR “personal trait*” OR “body image” OR satisfaction OR “time availability” OR “perceived environment” OR family OR peer* OR school* OR leader* OR coach* OR group* OR “climate” OR network* OR employment OR retirement OR “educational level” OR SES OR “socioeconomic status” OR “local identity” OR “national identity” OR value* OR tradition* OR “social expectation*” OR “social trend*” OR “social barrier*” OR “availability of tool*” OR “availability of service*” OR “access to tool*” OR “access to service*” OR neighborhood OR “community route*” OR “school environment” OR “work environment” OR architecture OR urbanization OR transport OR traffic OR “facilit* in public space*” OR advertisement OR “availability of sport club*” OR “availability of fitness center*” OR advocacy OR lobbying OR “corporate social responsibility” OR “physical activity promotion initiative*” OR legislation OR health OR education OR tourism OR environment OR “urban planning” OR transport* OR sport OR sports OR culture OR dance OR theater OR “gender mainstreaming” OR “social inclusion” OR “fiscal measure*” OR program* OR plan OR plans OR communication OR media OR guideline*
#4	“systematic literature review” OR “meta-analysis”

SLRs or MAs of observational primary studies on the association between any determinants and PA, Ex, or sport as the main outcome, were included in the umbrella SLR. The following SLRs and MAs were excluded: i) SLRs and MAs of intervention studies; ii) SLRs and MAs that focused on specific population groups (e.g. people with chronic diseases); iii) umbrella SLRs on the same topic (e.g. reviews of SLRs or MAs of epidemiological studies on variables in association with PA). Psychological determinants were defined as the person’s individual characteristics, such as cognitions, beliefs and motivation, that could potentially be associated with PA [4].

### Selection process

Following the systematic literature search, identified articles were arranged alphabetically and distributed among the partners belonging to the DEDIPAC-KH. Two reviewers independently screened the titles, abstracts, and full texts of assigned articles and assessed them for eligibility. Before final inclusion or exclusion, a common decision had to be reached for each SLR and MA; any uncertainty and disagreement was resolved by consulting three further authors (SB, LC, AP).

### Data extraction

A predefined data extraction form, developed by the DEDIPAC-KH and checked by two authors (KA, AP), was used to extract data for each included review. In reporting data, authors agreed to define as ‘reviews’ those SLRs and MAs found eligible for the umbrella SLR, and as ‘primary studies’ those studies included in the eligible SLRs and MAs. Moreover, authors agreed to consider all of the terminologies and forms of PA, including unstructured (i.e., PA linked with daily life) and structured (i.e., Ex and sports), independently from their frequency, duration, and intensity. Sedentary behavior was not included in the DEDIPAC umbrella SLRs.

The following information was extracted from each included review: year of publication, type of review (either SLR or MA), number of eligible primary studies included in this psychological umbrella SLR over the total number of studies included in the review; continent/s, primary study design, overall sample size, age range or mean age, gender proportion (% female), and year of publication of eligible primary studies; PA outcome details, type of determinant/s, aim of the review; overall results (qualitative or quantitative), overall recommendations, and limitations as provided by the review itself.

### Evaluation of importance of determinants and strength of the evidence

The results retrieved from the eligible primary studies included in the reviews were summarized by combining two slightly modified grading scales, previously used by the World Cancer Research Fund [22] and Sleddens et al. [23]. According to Sleddens [23], the codes + and ++ were used if there is an association (no matter if positive or negative). This was modified for the present review to report both the association and the direction of the association according to a recent umbrella SLR on behavioral determinants of PA [21].

The first scale grades the importance of a determinant referring only to the consistency and direction of the associations among the individual eligible primary studies. The importance was scored a (—) if all identified eligible primary studies, without exception, reported a negative association between the determinant and the outcome and a (-) if the negative association was found in more than 75% of the eligible primary studies. The importance of the determinant was scored a (0) if the results were mixed, or more specifically, if the variable was found to be a determinant and/or reported an association (either positive or negative) in 25% to 75% of eligible primary studies. Furthermore, the importance of the determinant scored a (+) if a positive association was found in more than 75% of the eligible primary studies and a (++) if a positive association was found in all eligible primary studies, without exception.

The second scale grades the strength of evidence, referring to the study design used among individual primary studies. The strength of the evidence was described as ‘convincing’ (Ce) if it was based on a substantial (n>10) number of longitudinal observational studies showing associations between the determinant and PA. The strength of the evidence was defined as ‘probable’ (Pe) if it was based on at least two cohort studies or five cross-control studies showing associations between the determinant and PA. Furthermore, the strength of the evidence was given as ‘limited suggestive evidence’ (Ls) if it was based mainly on findings from cross-sectional studies showing associations between the determinant and PA. Evidence was labelled as ‘limited, no conclusive evidence’ (Lnc) if the study findings were suggestive but insufficient to provide an association between the determinant and PA (and if no longitudinal data available).

### Quality assessment

The methodological quality of the included reviews was assessed using a modified version of the AMSTAR Checklist [24]. One of the criteria (question number 11) referring to the presence of any conflict of interest, was modified after a consensus between the DEDIPAC-KH partners, so that the conflict of interest was evaluated in the reviews and not in the primary studies included in each review.

Using the same methodology as Sleddens et al. [23], the included reviews were independently evaluated by two authors belonging to the DEDIPAC-KH. Any uncertainty and disagreement was resolved by consulting three further authors (SB, LC, AP). The eleven criteria were evaluated and scored 1, when the criterion was applicable to and fulfilled by the analyzed review or 0, when the criterion was not applicable to or not fulfilled by or could not be answered based on the analyzed review. Consequently, the sum quality score for each included review ranged from 0 to 11. The quality of the review was labelled as weak (0–3 quality points), moderate (4–7 quality points), or strong (8–11 quality points).

## Results

### SLRs and MAs selection process

As summarized in Fig 1, the systematic search identified 17,941 reviews that were potentially relevant for inclusion in the seven umbrella SLRs of the DEDIPAC-KH. After the removal of duplicates, 15,147 reviews remained for screening. After title and abstract reading, 12,414 (concerning title) and 2,733 (concerning abstract) reviews, respectively, were excluded because they did not meet the inclusion criteria. Thus, a total number of 535 full-text reviews were assessed for eligibility. After the full-text reading phase, the final number of SLRs and MAs eligible for the seven umbrella SLRs was 63. Of these, 43 reviews did not examine psychological determinants of PA. Therefore, the final number of reviews included in this umbrella SLR was 20 (14 SLRs and 6 MAs).

### Characteristics of the reviews and quality assessment

The characteristics of the 20 included reviews are summarized in Table 2.

**Table 2:** Characteristics of the included reviews (n = 20).

Author, Date (Type of review) [Ref]	Number of eligible primary studies included in the umbrella review/total number of primary studies included in the review	Continent/s of eligible primary studies	Study design of eligible primary studies	Total sample size of eligible primary studies (sample range)	Age range or mean (years) of eligible primary studies	Gender (female, % range) of eligible primary studies	Year range of eligible primary studies
**Babakus WS, 2012** **(SLR) [41]**	7/38	Europe (n = 5) Australia (n = 1) North America (n = 1)	Qualitative (n = 7)	398 (8–114)	26–83	25–100	2006–2012
**Babic MJ, 2014** **(MA) [25]**	99/111	Europe (n = 58) North America (n = 27) Australia (n = 8) Asia (n = 3) South America (n = 2) Africa (n = 1)	Cross-sectional (n = 77) Longitudinal (n = 22)	90,362 (46–10,987)	5–20	25–100	1991–2013
**Barnett I, 2012** **(SLR) [26]**	1/19	North America (n = 1)	Cross-sectional (n = 1)	2,899	57–71	0	1985
**Bui L, 2011** **(SLR) [27]**	8/20	North America (n = 4) Europe (n = 2) Australia (n = 2)	Prospective (n = 3) Cross-sectional (n = 3) Longitudinal (n = 2)	8,290 (76–1,602)	18–75	47–100	2002–2009
**Craggs C, 2011** **(SLR) [39]**	25/46	North America (n = 20) Europe (n = 3) Asia (n = 1) Australia (n = 1)	Prospective (n = 25)	33,196 (132–12,812)	4–9 10–13 14–18	N.A.	1986–2010
**Koeneman MA, 2011** **(SLR) [42]**	6/34	N.A.	Observational (n = 6)	N.A.	54–85	N.A.	1999–2008
**McDermott, 2015** **(MA) [44]**	84/84	N.A.	N.A.	N.A. (22–1,582)	18–65	N.A.	1987–2014
**Nasuti G, 2013** **(MA) [28]**	40/70	North America (n = 22) Europe (n = 12) Asia (n = 5) Australia (n = 1)	Cross-sectional (n = 32) Prospective (n = 8)	32,705 (30–6,078)	9–18	N.A.	1990–2011
**Olsen JM, 2013** **(SLR) [29]**	16/21	N.A.	Cross sectional (n = 10) Cohort (n = 1) Qualitative (n = 5)	10,371 (17–2,338)	19–75	100	2000–2010
**Owen KB, 2014** **(MA) [30]**	44/46	Europe (n = 27) North America (n = 8) Asia (n = 5) Australia (n = 1) Multiple Continents (n = 1) N.A. (n = 2)	Cross sectional (n = 26) Prospective (n = 13) Longitudinal (n = 5)	15,980 (61–1,071)	5–18	0–79	1992–2013
**Pavey T, 2012** **(SLR) [43]**	3/20	Mainly Europe	Observational (n = 3)	198 (30–49)	51–64	57–100	2005–2008
**Rhodes RE, 2006** **(MA) [31]**	34/34	North America (n = 17) Europe (n = 9) Asia (n = 1) Africa (n = 1) N.A. (n = 6)	Cross-sectional (n = 18) Prospective (n = 12) Longitudinal (n = 4)	63,380 (35–22,448)	19–77	0–100	1969–2006
**Ridgers ND, 2012** **(SLR) [32]**	3/53	North America (n = 1) Europe (n = 2)	Cross-sectional (n = 3)	N.A.	5–18	N.A.	2003–2010
**Siddiqi Z, 2011** **(SLR) [33]**	25/29	N.A.	Cross-sectional (n = 25)	986 (14–89)	18–89	45–100	1995–2009
**Stanley RM, 2012** **(SLR) [34]**	3/22	North America (n = 2) Australia (n = 1)	Cross-sectional (n = 2) Questionnaire Validation Study (n = 1)	N.A.	8–14	N.A.	1997–2010
**Stults-Kolehmainen MA, 2014** **(SLR) [35]**	168/168	N.A.	Cross-sectional (n = 100) Prospective (n = 55) Qualitative (n = 9) Retrospective (n = 4)	495,915 (9–46,573)	7–92	0–100	1980–2012
**Teixeira PR, 2012** **(SLR) [36]**	56/66	N.A.	Cross-sectional (n = 43) Prospective (n = 11) Mixed methods (n = 2)	26,540 (40–1,572)	18–65	0–100	1990–2011
**Uijtdewillingen L, 2011** **(SLR) [40]**	10/30	North America (n = 6) Europe (n = 3) Multiple Continents (n = 1)	Prospective (n = 10)	18,875 (155–12,812)	4–12 13–18	51–100	2006–2010
**Van der Horst K, 2007** **(SLR) [37]**	33/57	N.A.	Cross sectional (n = 29) Prospective (n = 4)	N.A.	4–12 13–18	N.A.	1999–2005
**Wilson KE, 2015** **(MA) [38]**	64/64	North America (n = 35) Europe (n = 22) Asia (n = 4) Australia (n = 2) South Africa (n = 1)	Cross sectional (n = 43) Prospective (n = 18) Mixed methods (n = 3)	88,400 (25–35,165)	15–93	0–100	1971–2013

Notes: MA: Meta-Analysis; N.A.: Not applicable; SLR: Systematic Literature Review.

Most of the reviews included eligible primary studies from multiple continents, mostly from Europe, North America, and Australia. Cross-sectional study design was used in the majority of eligible primary studies [25–38]. Thirteen reviews included retrospective, prospective and cohort studies, either as the only eligible study design [39–40] or as part of the eligible primary studies [25–31, 35–38]. One review included only qualitative studies [41], two other only observational studies [42, 43] while no information about the study design was presented in one review [44].

In five reviews, it was not possible to retrieve the total sample size of the eligible primary studies [32, 34, 37, 42, 44]. In the remaining studies, the total sample size ranged from 198 [43] to 495,915 [35]. Eight reviews referred to eligible primary studies including only young people. Among these, children and adolescents (≤18 years) were assessed in seven reviews [28, 30, 32, 34, 37, 39, 40]. Ten reviews considered adults [26, 27, 29, 31, 33, 36, 41–44], while two reviews considered the whole age range from youth to old age [35–38]. As some of the eligible primary studies included only one gender sample, the percentage of female participants ranged from 0 to 100%, though that data was absent in seven reviews [28, 32, 34, 37, 39, 42, 44].

### Measurements of PA

Among the 729 eligible primary studies included in this umbrella SLR, 567 studies from eighteen reviews used non-objective PA measurements (self-report, parental/teacher report, questionnaire) [25–32, 34–43]. Objective measurements of PA, either assessed by accelerometers, pedometers, heart rate monitors or direct observation, were used in 44 of the eligible primary studies, included in eight reviews [25, 28, 30, 32, 36–39]. Seven eligible primary studies included in four reviews combined objective with non-objective measures of PA [27, 28, 36, 39]. Finally, 111 eligible primary studies from four reviews did not report PA measures [29, 33, 43, 44].

The majority (n = 13) of the included reviews evaluated overall PA as an outcome [25, 28, 30, 31, 33, 35, 37–42, 44]. Three reviews measured time-specific PA: school break time PA and after school PA [34], recess PA [32] and PA changes across transition to retirement [26], which were considered as overall PA. Three reviews measured PA intention and/or behavior [27, 29, 36], and two the combination of PA and Ex (overall PA/Ex) [35, 42]. Lastly, one review measured a specific Ex referral schemes adherence (ERS) [43], which was considered as overall Ex. For all data (outcome, determinant, review aim, overall qualitative and quantitative results, limitations and recommendations) of the included reviews see the S1 Table.

### Categorization of the included determinants

After extraction of included reviews, a total number of 84 psychological determinants of PA were identified. In case of synonyms/duplicates or equally defined determinants, groups were arranged and labelled. For example, the determinants ‘perceived competence’, ‘competence’, ‘athletic competence’, and ‘sport competence’ were merged into the determinant ‘perceived competence’. Differently, other similar determinants were identified to form a sub-group with a specific label. For example, the determinants ‘affective judgment’, ‘enjoyment’, ‘interest’, ‘positive affect’, ‘psychological wellbeing’, and ‘satisfaction’ were part of the sub-group *Emotions and feelings*. After achieving a final consensus among authors, the final number of psychological determinants was 61, organized into the following categories: *Basic psychological needs*, *Emotions and feelings*, *Domain-general/specific perceptions*, *Motivation*, *Personal dispositions and cognitive skills*, *Personality traits*, *Perceived barriers/Adverse responses*, *Perceived benefits of PA*, *Psychological distress and disorders*, and *Rewards*. The categorization of determinants has been decided after their identification, to reduce the total number of them and to better evaluate their importance and strength of the evidence. Since the aim of this umbrella SLR was to provide a systematic overview of the psychological determinants of PA, the approach adopted by the authors was to consider the existing determinants and to analyze how they were associated with PA. Since the goal was not to describe or interpret the determinant, the authors did not interfere with the name given to a specific determinant and with its possible meaning.

### Findings of the reviews

Table 3 summarizes the importance and evidence of the associations between psychological determinants and different types of PA different age groups.

**Table 3:** Summary of the results of the included reviews: The importance of a determinant and its strength of evidence.

Determinant	Children 4–13 (Overall PA)	Adolescents 14–18 (Overall PA)	Children and adolescents 4–18 (Overall PA)	Adults 18–40 (Overall PA)	Adults >40 (Overall PA)	Adults >40 (Overall EX)	Adults >40 (Overall PA/EX)	Rural women >18 (PA behavior)	All ages ≥7 (Overall PA, intention, and behavior)	All ages ≥7 (Overall PA/EX)
**Basic psychological needs**										
*Autonomy*						0, Lnc [43]			0, Ls [38]	
*Competence*									+, Pe [38]	
*Relatedness*									0, Ls [38]	
**Emotions and feelings**										
*Affective judgment*			+, Pe [28]							
*Enjoyment*	0, Ls [37,39]	0, Ls [37,39]	0, Lnc [32,34]						+, Ls [33]	
*Interest*	0, Lnc [39]		+, Ls [32]							
*Positive affect*								+, Lnc [29]		
*Psychological wellbeing*						0, Lnc [43]			+, Ls [33]	
*Satisfaction*	0, Lnc [39]	0, Lnc [39]	* *			0, Lnc [43]				
**Domain-general/specific perceptions**										
*Perceived behavioral control*	0, Ls [39,40]	+, Pe [39,40]								
*Perceived physical appearance*			+, Pe [25]							
*Perceived fitness*			+, Pe [25]							
*Perceived vulnerability*			* *						-, Lnc [27]	
*Perceived competence*	0, Ls [39]	0, Ls [37,39,40]	+, Pe [25]							
*Physical self-concept*			+, Pe [25]							
*Self-acceptance*	0, Lnc [39]	0, Lnc [39]								
*Self-efficacy*	+, Ce [37,39]	+, Ce [37,39]	0, Lnc [34]		0, Lnc [42]	0, Lnc [42,43]	0, Lnc [42]	+, Ls [29]	+, Pe [27]	
*Self-esteem*	0, Lnc [39]	0, Lnc [39]								
*Self-perceptions*	0, Ls [37,39]	0, Ls [37,39]								
*Self-worth*	0, Ls [39]	+, Ls [39]								
**Motivation**										
*Autonomous motivation/regulation*			+, Pe [30]						+, Pe [36]	
*Intrinsic motivation*									+, Pe [36]	
*Extrinsic motivation*										
*Integrated regulation*									+, Ls [36]	
*Identified regulation*									+, Pe [36]	
*Introjected regulation*			0, Ls [30]						0, Ls [36]	
*External/controlled regulation*			0, Ls [30]						0, Ls [36]	
*Lack of motivation/Amotivation*			-, Ls [30]						-, Ls [33,36]	
*Exercise causality orientation*									0, Ls [36]	
*Motivation*		+, Ls [37]						+, Lnc [29]		
*Self-determination*			* *			0, Lnc [43]				
**Personal dispositions and cognitive skills**										
*Attitude*	0, Ls [39]	0, Ls [37,39,40]			+, Lnc [26]		0, Lnc [42]	+, Lnc [29]	+, Lnc [27]	
*Belief*		0, Lnc [40]	0, Lnc [34]	+, Lnc [41]	+, Lnc [42]					
*Expectation of change*						0, Lnc [43]				
*Goal setting/Planning*		++, Pe [39,40]	* *							
*Intention*	+, Pe [39,40]	0, Ls [37,39,40]					+, Lnc [42]		+, Pe [27,44]	
*Self-discipline*								+, Lnc [29]		
*Value*	0, Lnc [39]									
**Personality traits**										
*Agreeableness*									0, Lnc [31,38]	
*Conscientiousness*									+, Pe [31,38]	
*Extraversion*									+, Pe [31,38]	
*Modesty*									-, Lnc [41]	
*Neuroticism*									-, Pe [31,28]	
*Openness to experience/intellect*									+, Pe [31,38]	
*Psychoticism*									0, Lnc [31]	
**Perceived barriers/Adverse responses**										
*Barriers to PA*	0, Ls [37]	0, Ls [37]			-, Lnc [41]	0, Lnc [42]	-, Lnc [42]			
*Pain/fatigue/weakness*					-, Lnc [41]	0, Lnc [42]			-, Ls [33]	
**Perceived benefits of PA**										
*Knowledge of PA benefits*	+, Ls [39]									
*Lack of knowledge of PA benefits*					-, Lnc [41]				-, Ls [33]	
*Perceived quality of the program*							+, Lnc [42]			
*Physical and mental health perceptions*		0, Ls [37,39]							+, Ls [33,36]	
*Weight control/body care*		0, Lnc [40]		+, Lnc [41]					+, Ls [33,36]	
**Psychological distress and disorders**										
*Depression/depressive symptoms*	0, Ls [39]	0, Ls [37,39]					-, Lnc [42]			
*Emotional distress*					-, Lnc [42]					
*Fear of injuries/falling*					-, Lnc [41]		-, Lnc [42]	-, Ls [29]		
*Fear to go out alone*					-, Lnc [41]					
*Maturity fears*	0, Lnc [39]									
*Selfish to take PA*									-, Lnc [41]	
*Stress*										-, Ce [35]
**Rewards**										
*Lack of support*					-, Lnc [41]					
*Need supportive climate*									+, Pe [36]	
*Reward*	0, Lnc [39]									

Notes: Ce: Convincing evidence; Ex: Exercise; Lnc: Limited, no conclusive evidence; Ls: Limited, suggestive evidence; PA: Physical Activity; Pe: Probable evidence.

The most frequently studied determinants were ‘attitude’ (n = 7) [26, 27, 29, 37, 39, 40, 42], ‘self-efficacy’ (n = 7) [27, 29, 34, 37, 39, 42, 43] and ‘intention’ (n = 6) [27, 37, 39, 40, 42, 44]. However, most of the determinants were considered in one review only [25, 27–29, 31, 36, 39, 41–43].

#### Children

Three reviews investigated the psychological determinants of overall PA in children under 13 years of age [37, 39, 40]. ‘Intention’, ‘knowledge of PA benefits’, and ‘self-efficacy’ were found to be positively associated with overall PA. Only ‘self-efficacy’ showed a convincing level of evidence (+, Ce [37, 39]), while ‘intention’ and ‘knowledge of PA benefits’ showed a probable (+, Pe [39, 40]) or limited, suggestive evidence (+, Ls [39]), respectively. Inconsistent associations with overall PA emerged for the other determinants.

#### Adolescents

Three reviews examined the psychological determinants of PA for adolescents (14–18 years old) in relation to overall PA [37, 39, 40]. ‘Goal setting/Planning’ was positively associated with overall PA in all the eligible primary studies included in the reviews, without exception, with probable level of evidence (++, Pe [39, 40]). ‘Perceived behavioral control’ was positively associated with overall PA in more than 75% of the eligible primary studies with a probable level of evidence (+, Pe [39, 40]), while ‘motivation’ [37] and ‘self-worth’ [39], were positively associated with a limited suggestive level of evidence (+, Ls). Finally, ‘self-efficacy’ [37, 39] was positively associated with overall PA with a convincing level of evidence (+, Ce). No consistent association with overall PA was found for all the other determinants.

#### Children and adolescents

Five reviews [25, 28, 30, 32, 34] examined the psychological determinants of PA in children and adolescents combined (≤18 years old years old). PA showed positive associations with ‘perceived competence’, ‘perceived physical appearance’, ‘perceived fitness’, and ‘physical self-concept’ (+, Pe [25]); ‘autonomous motivation/regulation’ (+, Pe [30]); ‘affective judgment’ (+, Pe [28]), in more than 75% of the eligible primary studies with a probable level of evidence. A limited, suggestive level of evidence was found for a positive association of ‘interest’ and PA (+, Ls [32]). A negative association with PA emerged for ‘lack of motivation/amotivation’ [30] in more than 75% of the eligible primary studies included in this umbrella review with a limited, suggestive level of evidence (-, Ls). No consistent association with overall PA was found for the other determinants.

#### Adults

Six reviews examined psychological determinants of PA in adults (18–40 and >40 years old) in relation to overall PA [26, 41, 42], overall PA behavior [29], overall Ex and PA/Ex [42, 43]. Overall PA was positively associated with ‘attitude’ [26] and ‘belief’ [42] in more than 75% of the eligible primary studies with limited, no conclusive evidence (+, Lnc). Negative associations were found for ‘barriers to PA’, ‘lack of knowledge of PA benefits’, ‘pain/fatigue/weakness’, ‘fear to go out alone’, ‘fear of injuries/falling’, and ‘lack of support’ [41]; ‘emotional distress’ [42], with a limited, no conclusive evidence (-, Lnc). No consistent association emerged between overall PA/Ex and ‘self-efficacy’ (0, Lnc [42]). With respect to overall PA/Ex ‘intention’ and ‘perceived quality of the program’ [40] were positively associated in more than 75% of the included studies with limited, no conclusive evidence (+, Lnc). Conversely, overall PA/Ex was negatively associated with ‘barriers to PA’, ‘depression/depressive symptoms’, and ‘fear of injuries/falling’ [42], with limited, no conclusive evidence (-, Lnc). Finally, no consistent association emerged between overall Ex and ‘attitude’, ‘barriers to PA’, ‘pain/fatigue/weakness’ [42]; ‘autonomy’, ‘expectation of change’, ‘self-determination’, ‘psychological wellbeing’, and ‘satisfaction’ [43]; ‘self-efficacy’ [42, 43].

The only review examining psychological determinants of PA in adults >40 years old in relation to overall PA [41], showed a positive association for ‘belief’ and ‘weight control/body care’ in more than 75% of the eligible primary studies with a limited, no conclusive evidence (+, Lnc). One review examined psychological determinants in rural women [29], revealing a positive association of PA behavior with ‘motivation’, ‘attitude’, ‘self-discipline’, and ‘positive affect’ with a limited, no conclusive evidence (+, Lnc), and ‘self-efficacy’, with limited suggestive evidence (+, Ls). ‘Fear of injuries/falling’ showed a negative association with PA in more than 75% of the eligible primary studies with a limited, no conclusive evidence (-, Lnc [29]).

#### All ages

Eight reviews [27, 31, 33, 35, 36, 38, 41, 44] examined the psychological determinants of PA in individuals older than 7 years. Overall PA, intention and behavior was positively associated to ‘competence’, ‘intrinsic motivation’, ‘identified regulation’, ‘autonomous motivation/regulation’ and ‘need supportive climate’ [36]; ‘intention’ [27, 44]; ‘conscientiousness’, ‘extraversion’ and ‘openness to experience/intellect’ [31, 38], and ‘self-efficacy’ [27] in more than 75% of the eligible primary studies. A negative association emerged for ‘neuroticism’ [31, 38] with a probable level of evidence (+, Pe).

Positive association with overall PA, intention and behavior also emerged for ‘integrated regulation’ [36]; ‘physical and mental health perceptions’ and ‘weight control/body care’ [33, 36]; ‘enjoyment’ and ‘psychological wellbeing’ [33], with a limited suggestive evidence (+, Ls), while ‘attitude’ [27] showed a positive association with limited, no conclusive evidence (+, Lnc). A negative association with limited, suggestive evidence (-, Ls) emerged for ‘lack of motivation/amotivation’ [33, 36]; ‘lack of knowledge of PA benefits’ and ‘pain/fatigue/weakness’ [33]. Overall PA showed a negative association with ‘modesty’ and ‘selfish to take PA’ [41], and ‘perceived vulnerability’ [27], with overall PA, intention, and behavior with limited, no conclusive evidence (-, Lnc). Overall PA/Ex showed a negative association with ‘stress’ [35] in more than 75% of the eligible primary studies with a convincing level of evidence (-, Ce). No consistent association emerged for the remaining determinants.

### Evaluation of the methodological quality of the reviews

The results of the quality assessment using the AMSTAR checklist are reported in Table 4. Among the 20 included reviews, the majority (n = 16) were evaluated as being of moderate quality [25–27, 29, 30, 32–36, 38–42, 44], two were identified as weak [31, 37], and two labelled as strong [28, 43]. Three reviews [34, 43, 44] did not provide all characteristics of the primary studies (including the supplementary material available), while only one review provided a full list of included and excluded studies [43].

**Table 4:** Quality Assessment of the included reviews using the AMSTAR checklist.

Author, Date (Type of review) [Ref]	Was an 'a priori' design provided?	Was there duplicate study selection and data extraction?	Was a comprehensive literature search performed?	Was the status of publication (i.e. grey literature) used as an inclusion criterion?	Was a list of studies (included and excluded) provided?	Were the characteristics of the included studies provided?	Was the scientific quality of the included studies assessed and documented?	Was the scientific quality of the included studies used appropriately in formulating conclusions?	Were the methods used to combine the findings of studies appropriate?	Was the likelihood of publication bias assessed?	Was the conflict of interest included?	Sum quality score*	Quality of the review**
Babakus WS, 2012 (SLR) [41]	No	Yes	Yes	Yes	No	Yes	Yes	No	Yes	No	Yes	7	Moderate
Babic MJ, 2014 (MA) [25]	No	Yes	Yes	No	No	Yes	C.A.	N.A.	Yes	Yes	Yes	6	Moderate
Barnett I, 2012 (SLR) [26]	No	Yes	Yes	Yes	No	Yes	Yes	Yes	Yes	C.A.	No	7	Moderate
Bui L, 2011 (SLR) [27]	Yes	Yes	Yes	Yes	No	Yes	Yes	Yes	N.A.	No	No	7	Moderate
Craggs C, 2011 (SLR) [39]	Yes	Yes	No	No	No	Yes	Yes	Yes	N.A.	No	Yes	6	Moderate
Koeneman MA, 2011 (SLR) [42]	No	Yes	Yes	No	No	Yes	Yes	Yes	C.A.	Yes	Yes	7	Moderate
McDermott, 2015 (MA) [44]	C.A.	Yes	Yes	No	No	No	No	N.A.	Yes	Yes	Yes	5	Moderate
Nasuti G, 2013 (MA) [28]	No	Yes	Yes	No	No	Yes	Yes	Yes	Yes	Yes	Yes	8	Strong
Olsen JM, 2013 (SLR) [29]	Yes	No	Yes	No	No	Yes	Yes	No	N.A.	No	No	4	Moderate
Owen KB, 2014 (MA) [30]	No	No	Yes	No	No	Yes	Yes	Yes	Yes	Yes	Yes	7	Moderate
Pavey T, 2012 (SLR) [43]	Yes	Yes	Yes	No	Yes	No	Yes	Yes	Yes	Yes	Yes	9	Strong
Rhodes RE, 2006 (MA) [31]	No	No	Yes	No	No	Yes	No	N.A.	No	No	Yes	3	Weak
Ridgers ND, 2012 (SLR) [32]	Yes	C.A	Yes	No	No	Yes	No	N.A.	N.A.	N.A.	Yes	4	Moderate
Siddiqi Z, 2011 (SLR) [33]	Yes	No	Yes	No	No	Yes	Yes	Yes	N.A.	No	Yes	6	Moderate
Stanley RM, 2012 (SLR) [34]	No	Yes	No	No	No	No	Yes	Yes	N.A.	No	Yes	4	Moderate
Stults-Kolehmainen MA, 2014 (SLR) [35]	No	No	Yes	No	No	Yes	Yes	Yes	N.A.	No	Yes	5	Moderate
Teixeira PR, 2012 (SLR) [36]	No	N.A.	Yes	Yes	No	Yes	No	N.A.	N.A.	No	Yes	4	Moderate
Uijtdewillingen L, 2011 (SLR) [40]	Yes	Yes	Yes	No	No	Yes	Yes	Yes	N.A.	N.A.	Yes	7	Moderate
Van der Horst K, 2007 (SLR) [37]	No	Yes	Yes	No	No	Yes	No	N.A.	N.A.	No	No	3	Weak
Wilson KE, 2015 (MA) [38]	No	No	Yes	No	No	Yes	No	N.A.	Yes	Yes	Yes	5	Moderate

Notes: C.A.: Can't answer; N.A.: Not applicable.

*0 when the criteria was not applicable for the included review; 1 when the criteria was applicable for the included review.

**Weak (score ranging from 0–3); Moderate (score ranging from 4–7); Strong (score ranging from 8–11).

## Discussion

This umbrella SLR aimed to provide a summary of the evidence of the psychological determinants of PA across the life course, by evaluating the importance and strength of the evidence, and methodological quality of the SLRs and MAs included. To our knowledge, this is the first umbrella SLR that examined all the potential psychological determinants of PA across the life course, including 729 primary studies and 20 reviews (e.g., SLRs and MAs). The results may provide directions for future research strategies and for (sub)populations of inteterest for targeted intervention strategies.

Overall, the psychological determinats of PA have been predominantly analyzed in youth (eight reviews out of twenty) or in youth and adults combined (seven reviews out of twenty). This highlights the importance attributed to investigating and understanding PA behaviors of young people from a person-centered perspective focused on individual characteristics. Childhood is considered the most crucial period of the lifecycle to educate and promote long-lasting health enhancing active lifestyles [45] that will be maintained during adulthood [37], to decrease the risk factors for NCDs [1], and to counteract the new ‘pandemic’ phenomenon of inactive lifestyles [46, 47]. Therefore, understanding the psychological determinants of PA at the earliest stages of life is crucial for targeted interventions tailored to increase PA levels throughout the life course. Conversely, despite the aging of the population worldwide [48], research that focuses on the psychological determinants of PA behaviors in adults and elderly remains limited. Instead, for these age groups, there seems to be larger perceived relevance by the scientific community for environmental determinants as mobility policies and financial measures and regulation for PA and sport [5].

When addressing determinants of PA, SLRs and MAs usually listed factors regardless of their type, by using categories only related to broad aspects (i.e., biological, environmental, behavioral, political, socio-cultural, economical and psychological). Since in the current study 61 psychological determinants have been identified, a categorisation was proposed for a better understanding and more synthetic overview of how they might influence PA. This sizeable number of psychological determinants might reflect the importance and complexity of the psychological mechanisms that underpin PA behaviors.

Convincing and probable evidence emerged for psychological determinants in the categories of *Basic psychological needs*, *Emotions and feelings*, *Domain-general/specific perceptions*, *Motivation*, *Personal dispositions and cognitive skills*, *Personality traits*, *Psychological distress and disorders*, and *Rewards*. Conversely, no consistent association could be found for the determinant in the categories of *Perceived barriers/Adverse responses* and *Perceived benefits of PA*.

The majority of the determinants in the current study belong to the *Personal dispositions and cognitive skills* category, with eleven reviews out of 20 analyzing the individual’s intention to perform a given behavior [49]. The present umbrella SLR noted a probable positive association between ‘intention’ and overall PA levels in both children [39, 40], and all ages [27, 44], and ‘goal setting/planning’ was consistently found to be probably positively associated with PA in adolescents only [39, 40]. This age specificity for adolescence is not surprising, since goal setting is a cognitive life skill [50] strongly relying on higher-level cognition as planning [51], whose window of opportunities for development extend into late adolescence [52]. Among the theoretical frameworks that propose the psychological processes involved in behavioral change, the theory of planned behavior (TPB), extension of the theory of reasoned action (TRA), appears to be prominent in the current umbrella SLR [49]. According to the TPB, human behavior is guided by beliefs about its consequences (e.g., behavioral beliefs), about the normative expectation of other people (e.g., normative beliefs), and about the extent to which the behavior is perceived as being under the own control or dependent on external factors (e.g., control beliefs) [53]. In testing the links between beliefs, attitudes, intentions and PA behavior, the TPB substantiates that intentions are predicted best by attitudes and perceived behavioral control, and less by subjective norms [13]. Moreover, the degree to which perceived behavioral control influences the behavior directly (rather than indirectly through intention) is hypothesized to depend on the degree of actual control over the behavior [54]. This implies that intention is more likely to find expression in behavior if the behavior is under volitional control, representing people’s actual control over the behavior [55].

In the category of *Personality traits*, a positive association with overall PA emerged for ‘conscientiousness’, ‘extraversion’, ‘openness to experience/intellect’, with a probable level of evidence in all ages [31, 38]. On the contrary, ‘neuroticism’ emerged to be probably negatively associated with overall PA, intention and behavior in all ages [31, 38]. It appears that individuals who score high on neuroticism exhibit high levels of anxiety, vulnerability, distress, depression and self-consciousness, which may reduce the opportunities to be physically active [31, 38]. Therefore, actions towards increasing emotional stability levels in those individuals scoring high on neuroticism may have a positive impact on their PA. However, psychologists generally agree that behavioral action is unlikely to arise directly from personality, rather personality is thought to influence behavioral perceptions, expectations and cognitions [31]. In consideration of the overall strength and consistency of the association linking PA behavior to intentions and selected personality traits (i.e., extroversion, conscientiousness, neuroticism), we could speculate that those traits possibly influence attitudes and a sense of control over engaging in PA, which in turn may influence PA through intention. Further research at the intersection point between the TPB and nomothetic/idiographic personality traits theories is warranted to explore potential interrelations and design PA behavior change strategies that capitalize on the potential mediating role of modifiable intentions in the relationship between personality traits and Ex behavior [56].

Several reviews (45%) explored the determinants of PA included in the category of *Domain-general/specific perceptions*. Among them, ‘self-efficacy’, defined as the ‘people’s beliefs about their capabilities to exercise control over their own level of functioning and over events that affect their lives’ [57], showed a convincing positive association with overall PA in children [37, 39] and adolescents [37, 39], and a probable positive association with overall PA, intention and behavior in adults [27]. Self-efficacy has been shown to be effective in predicting and facilitating PA participation and Ex adoption, highlighting the importance of enhancing the one’s own efficacy perception to promote positive PA behaviors. In line with that, ‘perceived behavioral control’, a variable congeneric to self-efficacy showed a positive probable association in adolescents [39, 40], confirming that the perceived ease of performing the behavior is crucial when determining PA. However, the reciprocal nature of the relationship between efficacy perceptions and behavior has to be taken into consideration, as the behavior will not be undertaken unless efficacy perceptions are sufficient [13]. Based on the strength and consistency of the above evidence, a promising intersection point to further our understanding of the psychological determinants of PA is that between TPB and self-efficacy theory, with a complementary contribution of self-efficacy and behavioral control perceptions to the intention to be physically active and actual PA behavior [58, 59].

Within the *Motivation* and *Basic psychological needs* categories, a probable positive association emerged for ‘intrinsic motivation’, ‘identified regulation’, and ‘competence’ in all ages [36], while ‘autonomous motivation/regulation’ was associated with overall PA in both children and adolescents [30] and adults [36]. According to the self-determination theory (SDT), the innate psychological needs and the conditions that foster the needs for competence, relatedness, and autonomy, are essential to facilitate optimal functioning, positive psychosocial development and well-being [60–62]. Research showed that motivation for PA is likely to be more robust if the choice of actions is self-determined which is likely to lead to a greater sense of well-being [63]. Based on these motivational outcomes of the umbrella review, a promising frontier for future interventional research might be the application of life skills interventions in PA and sports that, by generating a motivational climate that is competence- and autonomy-supportive, may lead to the satisfaction of the corresponding basic needs and, finally, to an active lifestyle and optimal psychological well-being [61].

Among the determinants included in the *Psychological distress and disorders* category, ‘stress’ emerged to be negatively associated to overall PA/Ex in all ages, with convincing evidence [35]. The majority of the primary studies supported the hypothesis that higher habitual stress is associated with lower levels of PA, and highlighted the theoretical, practical, and clinical role of stress in PA behaviors [35]. Conversely, some prospective studies (18%) report evidence that PA was positively impacted by stress, as some individuals might utilize exercise to cope with stress. Therefore, further prospective study designs are recommended to investigate the relationship between stress and PA, in addition to the strategies that can potentially alleviate psychological stress in individuals.

In the *Emotions and feelings* category, ‘affective judgment’, defined as ‘the overall pleasure/displeasure, enjoyment, and feeling states expected from enacting an activity or from reflection on past activity’ [64, 65], was the only determinant associated with higher PA in children and adolescent with probable level of evidence [28]. The majority of theoretical models of PA include some aspect of affective judgment. In particular, the TPB distinguishes social outcome judgments (e.g., normative beliefs) from personal ones (e.g., attitude) [49]; social cognitive theory includes social, physical, and self-evaluative outcome judgments [66], while positive and negative outcome judgments are discussed in the transtheoretical and health belief model (HBM) [67, 68]. Finally, the SDT [60] highlights the importance of affective judgment in the intrinsic regulation and autonomous motivation. Moreover, research showed that PA participation could be predicted from positive affective responses to acute bouts of Ex [69, 70], confirming that affect could be an important determinant of PA [71]. Since cognitive and emotional self-regulation rely on intertwined brain substrates and functions that come into play in PA behaviors [72], we suggest the relevance, for future research, of investigating the joint role of the psychological determinants of PA behaviors falling into the categories of cognitive skills and emotions.

Determinants included in the *Perceived benefits of PA* and *Perceived barriers/Adverse responses* categories were positively or negatively associated with PA, respectively, with limited, suggestive or no conclusive evidence, or were not consistently associated, suggesting that the potential positive/negative consequences of a specific action [73] are not considered crucial, or strong enough, to promote long-lasting active lifestyles.

This umbrella SLR provided knowledge about the associations between psychological determinants and PA. Although a notable amount of SLRs and Mas and primary studies have been undertaken, some limitations need to be considered as they might have influenced the overall analysis and conclusions drawn in this umbrella SLR. The first concern relates to the need of clear definitions of PA behaviors to prevent confusion and difficulty in interpretation of homonymous terms [3, 74]. The present study was based on the consensus of the DEDIPAC-KH research team (consisting of 23 participants from five partner Nations) on a common nomenclature for PA that encompasses any bodily movement produced by skeletal muscles that results in energy expenditure, which may be unstructured and everyday life activity, exercise that includes prearranged, deliberate, and repetitive activity, and grassroots sports and competitive sports [5]. Despite this comprehensive definition ensures an extensive perspective of determinants of PA behaviors, it does not guarantee that the authors of the considered SLRs and Mas adopted the same pragmatic approach. The second concern pertains the absence of clear and well-established definitions used by scholars to summarize the information of determinants included in the primary studies they analyzed. To avoid misinterpretation of labels due to cultural biases, in the present umbrella SLR it was decided to refer to the actual terminology provided in the SLRs and Mas. The third concern refers to the difficulty in detecting information related to specific aspects of PA engagement, such as frequency and duration, and typology of exercise. Finally, a wide range of study designs, measurement techniques, population groups from countries with different cultural backgrounds, determinants investigated, and PA outcomes were included in the eligible primary studies, making it difficult to evaluate the evidence and draw definitive conclusions. To subsume cultural biases, to allow the possibility to obtain clear interpretations and generalizability of findings, and to hint at a way forward, scholars are urged to reach a consensus on clear definitions of relevant psychological determinants of PA. Indeed, the quality criteria and the condensed form of the current research can provide an important impetus for the further tackling these challenges. To note, cross-sectional studies were the most common study design, hence limiting the strength of the evidence for most of the determinants. Moreover, the majority of PA outcomes were obtained by non-objective measurement methods, which provide less accurate data for PA evaluation [75].

The current umbrella SLR has provided some insights into the psychological determinants that can potentially influence PA behaviors across the life course. To what extent these determinants are predictive of PA can perhaps be better understood through existing health behavior models and theories. Not only does this point to the importance of considering psychological mechanisms that might underpin PA behavior. Crucially, it highlights the complexity of the psychological determinants involved and indicates categories of determinants which, being supported by strong and consistent evidence and presenting potential intersection points, might be explored in combination in future studies to obtain a more comprehensive view. Therefore, future research should examine how the interactions between these determinants and psychological theories behind, might influence PA behaviors, leading to a theoretical integration that can further our understanding not only of multiple psychological predictors, but also of moderated and mediated prediction of PA behaviors.

In conclusion, the differences in the predictive value of the psychological determinants of PA seem to be most informative when referring to several theories and models used to explain human behaviors. Individuals’ beliefs, values, and goals, and how they relate to the achievement of behaviors, should be carefully taken into consideration and integrated with each other, to understand the mechanisms underlying the PA behaviors [55]. An attempt to integrate theories has been proposed in a theoretical model of motivation for physical education (PE) [76]. In particular, trying to explain the processes by which students’ autonomous motivation toward activities in PE lessons affects students’ participation in out-of-school leisure PA, a trans-contextual model of motivation has been developed [77]. The model incorporates specific aspects of SDT [60], a hierarchical model of intrinsic motivation [78], and TPB [49], providing a starting point for the development of a comprehensive model of behavior change, also including implicit processes and volitional planning. A theoretical development testing is therefore needed to explain phenomena, to better help people change their behavior, also in designing and delivering proper interventions [54].

As a final outlook, future research should zoom out to encompass a larger framework of multi-level determinants that are individual, interpersonal, or environmental in nature may interact in moderated or mediated ways with the psychological determinants of PA behaviors. In this respect, the European conceptual framework of PA determinants (EU-PAD) developed from cumulated experience of European scholars and policy makers [5] could illuminate on the relationship between multiple psychological factors and guide the development of a novel and integrated approach to investigate specific mechanisms and interactions for the implementation of active lifestyle behaviors of the individual.

## Supporting information

S1 Checklist PRISMA checklist. (DOC)

S1 Table Results of the included reviews. BMI: Body Mass Index; ERS: Ex Referral Schemes; ES: Effect Size; Ex: Exercise; LTPA: Leisure-Time Physical Activity; MA: Meta-Analysis; MVPA: Moderate to Vigorous Physical Activity; N.A.: Not Applicable; PA: Physical Activity; SLR: Systematic Literature Review.
(DOC)
