# Supplementary material for: Psychological determinants of physical activity across the life course: A "DEterminants of DIet and Physical ACtivity" (DEDIPAC) umbrella systematic literature review
Source: PLoS One. 2017 Aug 17;12(8):e0182709. doi: 10.1371/journal.pone.0182709 (PMC5560721; doi:10.1371/journal.pone.0182709)
Supplement: S1 Table — BMI: Body Mass Index; ERS: Ex Referral Schemes; ES: Effect Size; Ex: Exercise; LTPA: Leisure-Time Physical Activity; MA: Meta-Analysis; MVPA: Moderate to Vigorous Physical Activity; N.A.: Not Applicable; PA: Physical Activity; SLR: Systematic Literature Review. (DOC) [file pone.0182709.s002.doc]

|  |  |  |  |  |  |  |  |
| --- | --- | --- | --- | --- | --- | --- | --- |
| **S1 Table. Results of the Included Reviews.** | | | | | | | |
| **Author, Date  (Type of review) [Ref]** | **Outcome(s)** | **Determinant(s)** | **Review aim** | **Overall qualitative results of the review** | **Overall quantitative results of the review** | **Overall limitations of the study** | **Overall Recommendations** |
|  |  |  |  |  |  |  |  |
| **Babakus WS, 2012**  **(SLR) [41]** | Mixture of PA (total, LTPA, home, work, active commuting, energy expenditure, occupational, intensity, steps or physical inactivity) | Barriers to PA; Belief; Body care; Fatigue; Fear of injuries; Fear to go out alone; Lack of knowledge of PA benefits; Lack of support; Modesty; Selfish to take PA; Weakness; Weight control | To assess what is known about the levels of PA and sedentary time and to contextualize these behaviors among South Asian women with an immigrant background | South Asian women are less active than the other ethnic groups as well as compared to South Asian males; knowledge of PA and its benefits was found to be lacking among SA | N.A. | No standardized method for quality evaluation; lack of details from some of the included papers; measurement and definition of PA varied widely; publication and researcher bias possibility; significant heterogeneity among studies | More research should be dedicated to standardize objective PA measurement and to understand how to utilize the resources of the individuals and communities to increase PA levels and overall health of South Asian women; future research is needed to assess levels of ST and contextualize sedentary behaviors |
| **Babic MJ, 2014**  **(MA) [25]** | Overall PA | Perceived competence; Perceived fitness; Perceived physical appearance; Physical self-concept | To determine the strength, and potential moderators, of associations between PA and physical self-concept (general and sub-domains) in children and adolescent | PA, physical self-concept and its various sub-domains are significantly associated in children and adolescents; age and sex are key moderators of the association between PA and physical self-concept | Perceived competence was most strongly associated with PA (r=0.30), followed by perceived fitness (r=0.26), general physical self-concept (r=0.25) and perceived physical appearance (r=0.12) | Unpublished and studies published only in English were included; studies that examined the association between physical fitness and physical self-concept were not included; the definition and assessment of physical self-concept and subdomains was not consistent across studies; most of the studies are cross-sectional or longitudinal | Strategies to increase physical self-concept and sub-domains, particularly perceived physical fitness and competence, may have a role to play in promoting PA in young people; results highlight the importance of understanding the physical-self and its links to health-related behaviors in youth; further studies are needed to determine the mechanisms responsible for the effects of PA on physical self-concept |
| **Barnett I, 2012**  **(SLR) [26]** | PA changes across transition to retirement (Ex, LTPA, total PA) | Attitude | To examine changes and predictors of changes in PA across the transition to retirement; whether these changes vary by SES; what is known about predictors of changes in PA across the retirement transition | Ex and LTPA increased after the transition to retirement, whereas the findings regarding changes in total PA were inconclusive; men increase their PA more than women; lower SES is associated with a decrease and high SES with an increase in PA | N.A. | Evidence on predictors of change was scarce and methodologically weak; no language or country restrictions; published peer-reviewed journal articles as well as gray research literature were included; multidisciplinary approach contributed to the heterogeneity of the results and to the unfeasibility of meta-analysis | Further studies should include other measures of SES, appropriate and valid PA measures, apply clear and relevant defınitions of retirement, and study predictors of PA change across the transition to retirement; qualitative studies and longitudinal studies with longer follow-up are needed |
| **Bui L, 2011**  **(SLR) [27]** | PA intention and behavior | Attitude; Intention; Perceived vulnerability; Self-efficacy | To review studies in the PA domain that have utilized the protection motivation theory in explaining and predicting PA intentions and behavior | Self -efficacy appears to be the most effective in predicting and facilitating PA participation | N.A. | Restriction to English-written research, keywords and subject headings; use of volunteer samples, low response rates, use of self-reported measures; inconsistency of the operationalization or measurement of PA; some study reviewed measured PA intentions only and not behavior | Continuing to examine the extent to which manipulating particular protection motivation theory constructs can influence PA intentions and behavior, designing and testing protection motivation theory interventions tailored to specific stages of behavior change or target groups, such as at-risk populations |
| **Craggs C, 2011**  **(SLR) [39]** | Overall PA | Attitude; Depressive symptoms; Enjoyment; Goal setting; Intention; Interest; Knowledge of PA benefits; Maturity fears; Perceived behavioral control; Perceived competence; Physical and mental health perceptions; Reward; Satisfaction; Self-acceptance; Self-efficacy; Self-esteem; Self-perceptions; Self-worth; Value | To systematically review the published evidence regarding determinants of change in PA in children and adolescents | Inconclusive associations were reported for large proportion of the determinants examined; girls consistently reported larger declines in PA than boys in younger children; parental marital status was consistently shown not to be associated with change in activity; higher levels of self-efficacy were associated with smaller declines compared to lower levels of self efficacy in older children and adolescents | N.A. | Possibility of publication bias (included published studies only); heterogeneity in study samples, exposure and outcome measures included in this review; some studies draw data from the same cohorts; semi-quantitative reporting used in the review that limits the classification of the associations | Further research should include objective measures of PA and use previously validated questionnaires to assess the investigated determinants; more high quality research is needed in all age groups, especially in younger children; investigation into determinants of change should take into account of specifıc physical intensities such as minutes spent in moderate or vigorous PA |
| **Koeneman MA, 2011 (SLR) [42]** | Overall PA, Overall EX, Overall PA/EX | Attitude; Barriers to PA; Belief; Depression; Depressive symptoms; Emotional distress; Fear of falling; Intention; Pain; Perceived quality of the program; Self-efficacy | To systematically review determinants of PA and Ex among healthy older adults | Insufficient evidence for most associations between possible determinants and PA or EX | N.A. | There may be possibility of publication bias; a wide age range is applied that might have masked some of the differences between subsamples inside that population; they excluded some specific subsamples of the older population; overall low quality of the studies included | The determinants of PA need further study that include the use of objective measures of PA and Ex and valid and reliable measures of determinants |
| **McDermott, 2015**  **(MA) [44]** | Overall PA | Intention | To examine the relation between intention and PA behaviors and its moderating effects | A positive correlation between intentionand PA emerged; the correlation between intention and PA behavior decreases as the period of temporal separation between intention and behavior increases | Intention was positively associated to PA (r=0.51); temporal separation was a significant moderator of thethis relation (r=0.46) | Reported heterogeneity remained high suggesting the presence of other moderators; the included studies restrict the ability to track how the intention-behavior association develops beyond six months | To maximize predictive capacity of the intention-behavior relationship, researchers should focus on predicting PA behavior in the short term; it might be prudent to incorporate multiple ‘top ups’ of intervention elements targeting intentions over time; given the extent to which the magnitude of the intention-behavior association decreases over time, the utility of intention as a predictor of meaningful levels of PA appears questionable |
| **Nasuti G, 2013**  **(MA) [28]** | Mixture of PA (total, steps, MVPA) | Affective Judgment | To appraise the overall effect of affective judgment on PA in youth via MA and explore moderators of this relationship | The relationship between affective judgment and PA among youth would be within a meaningful ES. This relationship was invariant to study quality, design, country, and measure of affective judgment employed. Gender, measure of PA, and recruitment location emerged as significant moderators among correlational studies. Single-component interventions targeting affect show promise; and when implementing interventions with children, competing sedentary activities should be considered | Random-effects MAindicated an r=0.26 foraffective judgment and PA in youth | Findings are limited to databases, search terms, and time frame considered | Future work on affective judgment and PA should use gender-specific samples or analyses, and employ both direct and indirect measures of PA assessment. For self-reported PA measures, it would be worthwhile to delve into exactly what type of PA is being recalled. Intervention research on preadolescents with behavioral economic theory, measuring adherence of PA is warranted. Further, affective judgment should be targeted on its own and if it is not, mediation tests are absolutely necessary |
| **Olsen JM, 2013**  **(SLR) [29]** | PA behavior | Attitude; Fear of injuries; Motivation; Positive affect; Self-discipline; Self-efficacy | To identify factors that influence PA in rural women | Rural women have been found to be less active and experience more barriers to PA than urban women; PA determinants among rural women can be categorized according to personal, socio-economic, and physical environment factors | N.A. | Evaluation of data and analysis was done by one reviewer only; the terms 'rural' and 'PA' were inconsistently defined among studies; exclusion of articles studying women outside USA | Additional research that clearly defines and consistently applies the terms rural and PA is needed to strengthen knowledge in this area |
| **Owen KB, 2014**  **(MA) [30]** | Overall PA | Amotivation; Autonomous motivation/regulation; External/controlled regulation; Introjected regulation | To systematically review studies that assessed the association between self-determined motivation and PA levels in children and adolescents | Overall levels of self-determined motivation had a weak to moderate, positive associations with PA; autonomous forms of motivation had moderate, positive associations with PA, whereas controlled forms of motivation had weak, negative associations with PA; amotivation had a weak, negative association with PA | Self-determined (ρ=0.21-0.31), autonomous (ρ=0.27-0.38), controlled (ρ=−0.03-−0.17) forms of motivation and amotivation (ρ=−0.11-−0.21) were associated with PA | Most studies employed a cross-sectional design; this review did not explore the inter-relations between basic psychological needs satisfaction, motivation, and PA | Further testing of self-determination theory tenets in longitudinal and experimental studies is warranted; further study into the influence of needs satisfaction on PA is clearly warranted; investigations that examine the social factors that influence needs satisfaction, motivation, and PA are also needed |
| **Pavey T, 2012**  **(SLR) [43]** | ERS adherence | Autonomy; Expectation of change; Psychological wellbeing; Satisfaction; Self-determination; Self-efficacy | To quantify the levels of ERS uptake and adherence and to identify predictive factors | Women were more likely to begin an ERS but were less likely to adhere than men; older people are more likely to uptake and adhere | N.A. | High heterogeneity between included studies | Future PA promotion research would greatly benefit from a consensus definition of uptake and adherence |
| **Rhodes RE, 2006**  **(MA) [31]** | Overall PA | Agreeableness; Conscientiousness; Extraversion; Neuroticism; Openness to experience/intellect; Psychoticism | To understand the association between major personality treats and PA | Neuroticism, Extraversion and Conscientiousness were reliable correlates of PA with small ES, whereas Openness to experience/intellect, Agreeableness, and Psychoticism were not associated with PA; personality moderators of PA mode seem possible | Extraversion (r=0.23), Neuroticism (r=−0.11) and Conscientiousness (r=0.20) were identified as correlates of PA. Openness to experience/intellect and Agreeableness, and Eysenck’s Psychoticism trait were not associated with PA | Research is too limited to draw definitive conclusions about gender, age and culture interactions with personality and PA, but preliminary research suggests relative invariance | Future research using multivariate analyses, personality-channelled PA interventions, longitudinal designs and objective PA measurement is recommended |
| **Ridgers ND, 2012**  **(SLR) [32]** | Recess PA | Enjoyment; Interest | To examine the correlates of children’s and adolescent’s PA during school recess periods | Higher perceived encouragement from parents, peers, and the school as a whole was associated with higher self-reported PA levels during recess periods, particularly in adolescents; overall facility provision (i.e., sum of facilities available) was positively associated with PA; a positive association was found between unfıxed equipment and recess PA in children; boys are more physically active during recess, no association was found for BMI/central adiposity and grade level | N.A. | The majority are small-sized and cross-sectional studies; MA is difficult to obtain given the limited number of studies and the lack of consistency in correlates assessed; a range of PA measures have been used; different cut-points and observation systems may have influenced the strength of associations observed | Further research should investigate correlates of children’s and adolescents’ recess PA for variables that were not investigated frequently but indicated positive associations with PA; further research using objective measures is needed to determine adolescents’ recess PA levels; future research should examine whether increasing access to school facilities during recess periods increases PA levels in children and adolescents; further research is needed to determine whether specifıc types of equipment, or the overall availability of unfıxed equipment, are associated with higher levels of PA; future research should examine the correlates of boys and girls PA separately; More research is needed concerning correlates of PA in recess period, particularly in adolescents; schools to increase overall facility provision, unfıxed equipment and methods to increase social support, particularly by peers |
| **Siddiqi Z, 2011**  **(SLR) [33]** | Overall PA | Body care; Enjoyment; Fatigue; Lack of knowledge of PA benefits; Lack of motivation; Physical and mental health perceptions; Psychological wellbeing; Weight control | To systematically review the qualitative literature pertaining to impediments and enablers of PA participation among African Americans | To effectively promote PA among African Americans, targeted interventions will need to address impediments at multiple levels: i.e. individual impediments (e.g. self-efficacy, lack of time and money), social barriers (e.g. lack of childcare and social support) and environmentaldeterminants (e.g. crime in neighborhoods and lackof access to safe Ex facilities and parks). Programs should take into account the specific needs of subgroups within the African American community (e.g. hair maintenance issues in African American women) | N.A. | Possibility of publication bias; many included studies included only women | To effectively promote PA among African Americans, targeted interventions will need to address impediments at multiple levels |
| **Stanley RM, 2012**  **(SLR) [34]** | School break time PA; after-school PA | Belief; Enjoyment; Self-efficacy | To identify the correlates of children' PA (8-14years) occurring during the school break-time and after school periods | Boys and younger children tend to be more active during break-time and after-school; BMI in females negatively associated with after-school PA, age was negatively associated in school-break and after school; family affluence, access to a gym, access to four or more PA programs and the condition of a playing field were all associated with school break time PA in one study; access to loose and fixed equipment, playground markings, size of and access to play space and the length of school break time were all positively associated with changes in school break time PA in intervention studies; in the after-school period, gender (with boys again more active), younger age, lower body mass index (for females), lower TV viewing/playing video games and greater access to facilities were associated with higher levels of after-school PA in two or more studies, while parent supervision was negatively associated with females’ after-school PA in one study | N.A. | Small number of studies that vary in methodological aspects; possibility that some studies are missed during the search process; majority of cross-sectional studies; some studies stratified analyses by salient variables such as age, gender and intensity of PA, resulting in an over-representation of these studies in the review; the relatively narrow age range specified in the current review is a limitation | Future studies using a context specific approach should identify and report specific facilities relevant to the context in question, which will contribute to a clearer understanding of context-specific PA; future studies should choose measurement tools with appropriate psychometric properties; Need of high quality evidence upon which PA promotion in young people can be tailored to specific settings and contexts |
| **Stults-Kolehmainen MA, 2014**  **(SLR) [35]** | Overall PA/Ex | Stress | To review the literature investigating the influence of stress on indicators of PA and Ex | The results showed an inverse association of stress and PA behaviors | N.A. | Limited amount of experimental evidence; issues with PA measurement and sample characteristics; temporal aspects of stress research; publication bias may be present | There is a need for additional models and a theoretical framework that describe the non-linear, bi-directional and dynamic nature of stress and PA relationships; links between stress, coping style, perceptions of energy and fatigue, energy expenditure metabolism, amongst other factors should be integrated into conceptual models explaining obesity and physical health; models specifically examining recovery from stressors and sedentary behavior would be useful |
| **Teixeira PR, 2012**  **(SLR) [36]** | PA behavior | Amotivation; Autonomous regulation; Autonomy; Body care; Controlled regulation; Ex causality orientation; External regulation; Identified regulation; Integrated regulation; Intrinsic motivation; Introjected regulation; Need supportive climate; Perceived competence; Physical and mental health perceptions; Physical and mental health perceptions; Relatedness | To examine the empirical literature on the relations between key self-determination theory-based constructs and Ex and PA behavioral outcomes | The results show consistent support for a positive relation between more autonomous forms of motivation and Ex, with a trend towards identified regulation predicting initial/short-term adoption more strongly than intrinsic motivation, and intrinsic motivation being more predictive of long-term Ex adherence. Competence satisfaction and more intrinsic motives positively predict Ex participation across a range of samples and settings. Mixed evidence was found concerning the role of other types of motives (e.g., health/fitness and body-related), and also the specific nature and consequences of introjected regulation | N.A. | Heterogeneity of the samples in the majority of studies; unpublished studies, evidence from grey literature, and data from non-English publications were not included; the way in which results from each study were classified and quantified is somewhat arbitrary and subject to criticism and various interpretations; the distal effects of some variables on behavior is thought to be mediated by other intermediate variables; the definition of “behavioral variable” to describe the outcome of choice, lumping together self-report and direct measures of behavior, and also attendance and stages of change is clearly not without reproach | Future studies may come to identify significant moderating factors for the role of specific regulations on Ex adherence, such as age, gender, previous health conditions, or social norms and social desirability. Future studies would do well to include biological markers of successful Ex-related outcomes such as increased fitness and reductions in disease risk factors |
| **Uijtdewillingen L, 2011**  **(SLR) [40]** | Overall PA | Attitude; Belief; Intention; Perceived behavioral control; Perceived competence; Planning; Weight control | To summarize and update the existing literature on determinants of PA and sedentary behavior in young people | Moderate evidence for intention as a determinant of children’s PA, and age (ie, older children were more active), ethnicity (ie, being of African–American descent determined being less physically active) and planning as determinant of adolescent PA | N.A. | Included studies assessed overall PA only; used two databases only; the selected language of publication was English only | Future researches should be focused on determinants of child and adolescent sedentary behavior and on environmental determinants of PA in both children and adolescents, should use reliable and valid measures of both determinants as well as the actual behaviors and should conduct prospective studies of high methodological quality |
| **Van der Horst K, 2007**  **(SLR) [37]** | Overall PA | Attitude; Barriers to PA; Depression; Enjoyment; Intention; Motivation; Perceived competence; Physical and mental health perceptions; Self-efficacy; Self-perceptions | To summarize and update the literature on correlates of PA, insufficient PA, and sedentary behavior in young people | For adolescents positive associations with PA were found for gender (male), parental education, attitude, self-efficacy, goal orientation/motivation, physical education/school sports, family influences, and friend support; For children, the evidence was insufficient to draw conclusions about correlates of insufficient PA | N.A. | Publication bias may be present; possibility of missed studies as a result of the search strategy; the main outcome was overall PA without other classifications; mostly cross-sectional studies included; because of the variability, it was not possible to assess the overall strength of the associations | More prospective studies are needed and more research including children |
| **Wilson KE, 2015**  **(MA) [38]** | Overall PA | Agreeableness; Conscientiousness; Extraversion; Neuroticism; Openness to experience/intellect | To estimate the population correlations between common personality factors and PA and to examine whether they vary according to selected sample characteristicsand study features | Extraversion, Neuroticism, Conscientiousness, and Openness were related to PA, but not Agreeableness; an age dependent relationship between PA and Agreeableness emerged; effects were moderated by characteristics of the sample or study features | Extraversion (r=0.11), Neuroticism (r=−0.07), Conscientiousness (r=0.10) and Openness (r=0.03) were related to PA, but not Agreeableness (r=0.00) | Limited statistical power to detect interactions, particularly 3-way interactions, resulted from the relatively small number of studies that reported on common or similarly defined moderator factors for each trait; multiple 2-way interactions and diversity in the measurement and conceptualization of PA and personality among these studies support the possibility of more complex interactions for which there is insufficient evidence to test; inability to correct for measurement error and moderator variables | Future studies should use better measures of PA and prospective designs, adjust for statistical artifacts, and consider advances in the conceptualization of personality |
|  |  |  |  |  |  |  |  |
